# Fluoroscopy-Guided Motion Management in Particle Therapy: Evolution, Challenges, and AI-Enabled Opportunities

**DOI:** 10.3390/tomography12050066

**Published:** 2026-05-09

**Authors:** Feifei Li, Keith M. Furutani, Chris J. Beltran

**Affiliations:** Department of Radiation Oncology, Mayo Clinic, Jacksonville, FL 32224, USA; furutani.keith@mayo.edu (K.M.F.); beltran.chris@mayo.edu (C.J.B.)

**Keywords:** fluoroscopy, particle therapy, machine learning

## Abstract

The sharp dose gradients that underpin the dosimetric advantage of particle therapy over photon therapy can be undermined by the interplay effects due to intra-fraction motion in modern pencil beam scanning systems. Fluoroscopy-Guided Particle Therapy (FGPT) offers a promising path to improved motion management through real-time tracking of tumors or surrogate signals. The advent of flat-panel detector (FPD)-based technology has enabled tighter integration of fluoroscopy/fluorography into treatment units and accelerated clinical adoption and research, with commercial systems such as Hitachi’s Real-time Gated Particle Therapy (RGPT) now available. However, the need for implanted fiducial markers, with the associated invasiveness and risk of complications, limits the utility of RGPT to a few anatomic sites in selected patients. The full potential of FGPT, therefore, depends on reliable marker-less tumor tracking, which remains challenging because soft-tissue targets are obscured by overlapping anatomy along the X-ray path, leading to reduced reliability of traditional image-registration algorithms in the projection domain. Recent advances in deep learning and AI-driven image registration have renewed hope for overcoming these barriers, enabling real-time marker-less tracking for particle therapy. This review outlines the evolution of fluoroscopy technology from image intensifier (II) to FPD-based systems, summarizes historical and recent vendor-supported FGPT strategies, and surveys emerging AI-based algorithms in the literature. A general review of machine learning-based image registration is provided, challenges in generalizability and interpretability are highlighted, and potential paths toward reliable, clinically deployable FGPT are discussed.

## Simple Summary

Particle therapy delivers radiation that stops sharply at a chosen depth, sparing healthy tissue near the tumor. This precision can be undermined when the target moves during respiration, as in many lung, liver, and pancreatic tumors, where small displacements can cause underdosing of the tumor or unintended dose to adjacent organs. Fluoroscopy enables real-time imaging of the target during treatment and is therefore a promising imaging modality for motion-managed particle therapy. This review traces the evolution of fluoroscopy hardware from image intensifiers to modern flat-panel detectors integrated with proton therapy units, summarizes vendor-supported fluoroscopy-guided systems, and examines why reliable tracking still relies on implanted fiducial markers. We then survey emerging AI-based methods that could lead to marker-less tumor tracking using on-treatment fluoroscopy. Technical and clinical challenges are discussed.

## 1. Introduction

The clinical application of particle therapy traces its origins back to Robert Wilson’s seminal 1946 paper [1]. In the aftermath of World War II, physicist Robert R. Wilson left the Manhattan Project to join Harvard University, determined to seek peaceful applications of atomic energy. Wilson proposed a therapeutic usage of protons based on their unique physical property known as the Bragg peak—a phenomenon where charged particles deposit the majority of their energy at a precise, controllable depth before stopping completely. He argued that by manipulating this peak, clinicians could deliver high doses of radiation to deep-seated tumors while sparing the healthy tissue surrounding them—a revolutionary concept that effectively birthed the field of particle therapy. Wilson’s vision found its first clinical realization at his own institution: in 1975, Gragoudas and colleagues at Massachusetts Eye and Ear used the Harvard Cyclotron Laboratory to deliver the first proton-beam treatment for choroidal melanoma, an eye-preserving alternative to enucleation that remains a standard of care today [2]. The technique exploits the precision of the Bragg peak in its most demanding setting: curative doses must be deposited millimeters from the optic nerve, retina, and lens. The same approach now extends to ocular surface tumors at the dedicated 60–70 MeV proton beamlines that have continued the Harvard tradition, including the Centre Antoine-Lacassagne in Nice and the CATANA Centre at INFN-LNS in Catania, where 48–60 Gy RBE is delivered to conjunctival melanoma and conjunctival squamous cell carcinoma in only four daily fractions, with a 90-to-10% dose fall-off of approximately 1 mm at 3 cm depth [3,4]. Ocular proton therapy thus represents both the historical proof and the geometric extreme of the precision agenda that motivates particle therapy more broadly—an agenda that, as the next sections detail, becomes substantially harder to realize when the target lies deep within a moving thoracic or abdominal anatomy. As of May 2026, there are 128 particle therapy facilities in clinical operations worldwide [5].

The steep dose gradient of particle therapy is a double-edged sword. It enables superior normal tissue sparing compared to conventional photon radiotherapy, yet it also makes particle therapy inherently more sensitive to geometric uncertainties, especially those arising from motion. Unlike photon therapy, where radiation passes completely through the patient, protons and heavy ions stop at a specific depth that is highly sensitive to the density and composition of the materials traversed. For a typical photon treatment beam, a 1.0 cm depth variation beyond the depth of maximum dose leads to approximately 3% dose difference. In contrast, for a proton beam, the same depth variation near the distal edge can result in up to 90% dose error [6]. Any motion that alters the radiological path length can shift the stopping point, potentially causing the beam to underdose the tumor or overdose healthy tissue immediately beyond it. This is especially critical in the lung, where solid tumor motion leads to significant alteration of water equivalent path length (WEPL). Lung tumors can move up to 30 mm [7], and respiratory motion can lead to WEPL change of 20.4 mm near the heart [8]. The benefit of particle therapy diminishes without precise patient and tumor positioning.

The impact of organ motion on radiation therapy has been the subject of numerous reviews [9,10,11,12,13,14]. In the context of particle therapy, dosimetric uncertainties arise from the combination of three primary sources of motion. Inter-fraction motion: discrepancies in patient positioning and anatomy relative to the baseline planning CT that occur between treatment fractions; typical examples include patient weight fluctuations and tumor progression or regression. Intra-fraction motion: physical movement of the patient’s body or internal organs during the delivery of a single treatment fraction; typical examples are respiration, digestion, and organ filling. Beam scanning motion: the temporal movement of the particle beam relative to the tumor volume; this is intrinsic to delivery mechanisms such as modern pencil beam scanning (PBS) [15], which requires a finite amount of time to scan through the entire target, both laterally and depth-wise. Inter-fraction motion can be mitigated through improved immobilization, precise positioning systems, and adaptive re-planning. However, intra-fraction motion remains a significant challenge for radiation therapy. Respiratory motion is a major concern for therapy delivered to the thoracic and abdominal regions [11,16]. Because particle therapy dosimetry is uniquely sensitive to positioning and range uncertainties, robust motion management is even more critical to particle therapy as compared to conventional X-ray therapy [12,16].

Motion management for particle therapy depends heavily on the mechanism of beam delivery. In early proton delivery systems, beams were delivered via passive scattering (PS) [17]. In this method, the narrow proton beam from the accelerator is broadened laterally by a scattering system and shaped in depth using ridge filters or modulator wheels. The lateral spread occurs on a timescale that is effectively instantaneous, while spreading along the distal direction occurs within one modulator revolution (typically < 0.1 s) [16]. Because the beam is broad and continuous, motion management was historically handled through robust planning margins. Clinicians utilized the internal target volume (ITV) [18], expanding the beam aperture to encompass the tumor’s entire trajectory. However, unlike photons, protons are highly sensitive to variations in WEPL. To mitigate motion-induced range uncertainty, custom-milled plastic compensators were designed using a technique called “smearing”. Smearing involves physically modifying the compensator’s topography to ensure distal coverage remains adequate even as the tumor and heterogeneities shift. While this method is robust, it adheres to a “static dose cloud” approximation that is only partially valid, and it often leads to the irradiation of significant healthy tissue to ensure coverage.

The inability of passive delivery to shape the dose proximal to the target led to the development of pencil beam scanning (PBS) [15,17]. Pioneered at the Paul Scherrer Institute (PSI), PBS replaces broad scattering foils with dipole magnets that steer a narrow “pencil” beam to paint the dose spot-by-spot and layer-by-layer. PBS systems operate in three primary modes: discrete spot scanning [15], raster scanning [19], and line scanning [20]. In discrete scanning mode, the lateral beam scans a grid of discrete spots and the beam is turned off between spots, resulting a few milliseconds of dead time; in raster scanning modes, the lateral beam scans through the same grid but the beam remains on during transitions, leaving small transient dose in between spots; in line scanning mode, the lateral beam scans continuously in space and time, not bounding to any grid. In PBS, lateral scanning is rapid (5–20 m/s), but changing the beam’s penetration depth (energy switching) is significantly slower. For Hitachi ProBeatV’s synchrotron system, energy switching can take 1 to 2 s for single energy extraction (SEE) and 0.2 s for multiple energy extraction (MEE) [21]. The time scale involved in energy switching is of the same order of magnitude as the respiration period. Without synchronization between beam delivery and respiration, the beam may be delivered preferentially to certain respiratory phases—a phenomenon known as the interplay effect [22,23]. This “dynamic-on-dynamic” problem cannot be solved by static margins or compensator smearing; it requires real-time synchronization between the beam delivery and the patient’s anatomy changes to ensure dosimetric robustness [24].

To mitigate the interplay effect on a PBS delivery system, one can adopt a passive management scheme by adapting the treatment plan or beam delivery methods in a way not regulated by the state of the patient’s breathing. A widely adopted approach is to improve the treatment plan robustness through 4D planning [25,26,27,28,29,30,31]. In terms of PBS delivery, rescanning [12,32,33,34,35,36,37,38,39,40] is the dominant practice with variants such as layered rescanning, volumetric rescanning, or phase-controlled rescanning (PCR) [20,29,41,42,43]. The drawbacks of rescanning include increased treatment time, some degree of sensitivity to the timing with respect to tumor motion, and the inability to deliver very small doses accurately.

Motion management can also be accomplished with active management: either the patient breathing or beam delivery, or both are modified to synchronize with each other so that the uncertainty of the delivered dose can be reduced. Breath hold (BH) [44] and abdominal compression (AC) [45] are two main approaches to reduce breathing motion in particle therapy. BH and AC are not always tolerable by patients, in which case gating is an alternative [46]. Respiratory gating is defined as the synchronization of radiation delivery with the respiratory cycle, correlating the beam-on and beam-off states with the physical location of the tumor to ensure accurate targeting. While external respiratory gating systems such as the real-time position management (RPM) system, Respiratory Gating for Scanners (RGSC), and Surface Guided Radiation Therapy (SGRT) have been widely adopted to approximate this strategy, they fundamentally rely on the assumption that external surface motion correlates predictably with internal tumor position. This assumption, however, is frequently challenged by physiological discrepancies. Hanley et al. [47] observed instances where the diaphragm moved 38 mm while the chest wall moved only 2.5 mm, highlighting a potential disconnect in motion magnitude between external surrogates and internal anatomy. It was also demonstrated that 3D tumor motion often differs significantly from surface motion due to hysteresis and phase shifts [48]. While some studies suggest using the diaphragm as a superior surrogate, Cerviño et al. emphasized that its correlation with tumor position is not universal and must be verified in a strictly patient-by-patient fashion [49]. Therefore, an ideal gating approach should generate the gating signal directly from internal tumor motion to ensure that beam delivery is synchronized to the actual position of the target volume [50,51].

**Figure 1:**
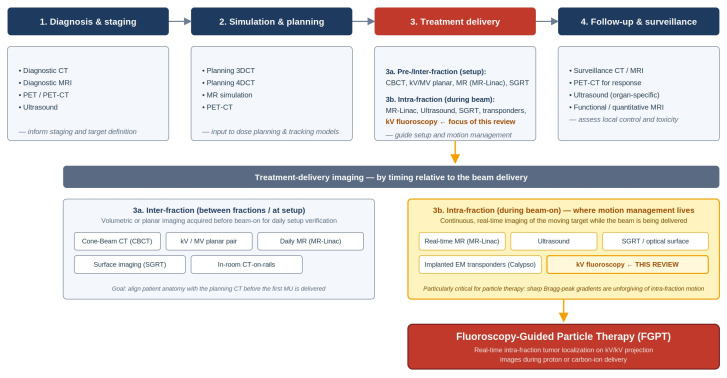
Imaging modalities commonly used across radiation therapy and the position of fluoroscopy-guided particle therapy (FGPT) as an intra-fraction motion management technique.

The benefit of active motion management is reported in a review by Riboldi et al., who summarized 12 studies involving 445 patients with lung, liver, and pancreatic cancers and concluded that real-time respiratory tumor tracking yields superior outcomes compared to those without [52]. Similarly, Zhang et al. found that integrated mega-voltage (MV) and kilo-voltage (kV) on-treatment tracking leads to a clinically significant reduction in late urinary toxicity [53]. While these successes in photon therapy underscore the potential of motion compensation, there remains an unmet clinical need in particle therapy, where the implementation of real-time tracking remains strikingly limited. Despite the heightened sensitivity of particle beams to anatomical motion, a 2023 worldwide survey revealed that only a small minority of particle therapy centers have successfully adopted either marker-based or marker-less real-time tracking [54]. While tracking based on internal signals is theoretically the “gold standard” for motion management, its scarcity in clinical practice highlights significant technical barriers and a clear opportunity for innovation. One promising avenue for addressing this gap is fluoroscopy-based tumor tracking, which can be implemented via both marker-based and marker-less approaches, and is the primary focus of this review. Figure 1 situates FGPT within the broader landscape of imaging modalities used across the radiation therapy patient pathway. The colored path traces the convergence that motivates this review—from intra-fraction motion management scheme at the treatment-delivery stage, through kV fluoroscopy, to fluoroscopy-guided particle therapy which Section 2, Section 3 and Section 4 develop in detail.

## 2. The Evolution of Fluoroscopy Imaging System

Fluoroscopy and radiography are both X-ray-based imaging techniques with an intricate, intertwined history, though they differ in their traditional output and application. Fluoroscopy produces real-time, continuous moving images on a screen, allowing clinicians to observe dynamic processes. Radiography, on the other hand, captures a single static photograph of the fluoroscopic image onto a medium. However, technological advances such as pulsed X-ray tubes and digital FPDs have brought these two modalities converging—modern systems can now seamlessly switch between real-time dynamic imaging and high-quality static image capture within a single digital platform, blurring the traditional distinctions between them [55].

The history of fluoroscopy and radiography is inseparable from the discovery of X-rays itself. In 1895, Wilhelm Röntgen became the first to observe real-time X-ray fluoroscopy when he watched the bones of his own hand moving between an X-ray source and a phosphor-coated screen. When he captured the now-famous radiograph of his wife’s hand and wedding ring, he produced the first radiograph. Thomas Edison subsequently refined the technology, discovering that calcium tungstate (CaW), as a phosphor material, produced brighter images than Röntgen’s barium platinocyanide. Edison developed a dedicated viewing device and named it the “fluoroscope”. This innovation enabled non-invasive visualization of internal anatomy and was rapidly adopted in medicine. However, the early era was marked by a dangerous lack of safety awareness. Unchecked enthusiasm led to inappropriate commercial applications, such as shoe-fitting machines in retail stores, and widespread unregulated exposure to ionizing radiation resulted in significant harm—including radiation burns and deaths among early operators and the public [56,57].

In the early generations of X-ray imaging, phosphor screens lacked the efficiency to produce adequate exposure rapidly; capturing an image on film required tens of minutes, rendering radiography impractical due to motion-induced blurring. Consequently, real-time fluoroscopy became the preferred diagnostic modality rather than radiography. However, despite the remarkable sensitivity of human vision, the luminance of early fluorescent screens was insufficient for daylight viewing. Clinicians were required to undergo a 30 min period of “dark adaptation” prior to procedures. This physiological necessity ensured the sensitization of retinal rod cells, which was essential for visualizing the low-luminance images produced by early fluoroscopic screens [56]. While rod-mediated (scotopic) vision is effective in low-light conditions, it suffers from significantly reduced visual acuity—approximately ten times lower than that of cone-mediated (photopic) vision. As a result, early fluoroscopy was characterized by poor image detail and often necessitated high radiation doses as physicians attempted to compensate for the dimness of the anatomical images.

A critical advance in fluoroscopy technology came in the 1950s with the introduction of the image intensifier (II)—a vacuum tube device that enabled fluoroscopy viewing in ambient light. The II is a chain of imaging components that converts X-ray photons to significantly intensified visible light photons [55], with one X-ray photon converted into several thousand visible light photons [58]. The process is done in stages along the imaging chain. First, X-ray photons strike an input scintillator material and are turned into visible light photons. The visible light photons then hit a photoelectric cathode and knock out photoelectrons that carry the visual signals. Major amplification is accomplished when photoelectrons are accelerated and focused by a high voltage of about 25,000 V [58], subsequently impinging onto an output phosphor material, generating a very bright visible light image. The first generation of II uses silver-activated zinc-cadmium-sulfide (ZnCdS:Ag) for the input phosphor material, but it was replaced by sodium-activated cesium-iodide (CsI:Na) in the mid-1970s. The introduction of CsI:Na doubled the scintillating efficiency of ZnCdS:Ag, owing to its higher X-ray stopping power; the required imaging dose is halved [56]. CsI remains the most common input scintillating layer on today’s flat-panel detector (FPD) based system. Prior to the emergence of FPD, the output of the II required optical coupling to viewing or recording devices. This was achieved through an optical distributor—a complex assembly of lenses and beam-splitting mirrors designed to direct the intensified light to various video components. The video capture technology itself underwent significant evolution, shifting from bulky analog vacuum tubes like the Vidicon and Plumbicon in the 1960s—which were prone to image lag and spatial distortion—to Charge-Coupled Devices (CCDs) in the 1980s [59].

By the 1990s, X-ray image intensifier technology had reached a high state of refinement with not much room for significant improvement in terms of Detective Quantum Efficiency (DQE) [56]. However, the device’s vacuum tube architecture remained subject to inherent physical constraints and artifacts [60]. Clinical image quality was compromised by “vignetting” (peripheral light loss) and “veiling glare” (a reduction in object contrast at the output phosphor caused by the internal scattering of light and electrons). Geometric fidelity was degraded by “pincushion distortion” which occurs because the X-ray beam is projected onto a curved input window, resulting in nonlinear magnification at the image periphery. Additionally, the electron optics were sensitive to external magnetic fields, altering electron paths to produce “S-distortion”. It was during this period that a new generation of detectors emerged, poised to replace the bulky image intensifier and its optical coupling with an integrated, compact digital system—the flat-panel detector (FPD). This technological shift was fueled by the development of large-area active-matrix liquid crystal displays. While initially intended for laptop computers, these arrays were validated as adequate for radiological applications as well [61,62,63,64]. FPDs became increasingly prevalent at the turn of the century. Their adoption enabled the standardization of digital radiography and eventually cone beam CT (CBCT), revolutionizing standard radiotherapy by transforming previously ‘blind’ treatments into high-precision, image-guided procedures [62,65,66,67,68].

The design of flat-panel detectors (FPD) is based on large-area active matrix arrays [61,69], typically constructed using hydrogenated amorphous silicon (a-Si:H) thin-film transistors (TFTs). Widely utilized in solar cell technology, a-Si is well-suited for use as a photodiode in large-area detectors; it demonstrates minimal radiation-induced degradation, a stability evidenced by its ability to withstand light intensities in solar applications that are orders of magnitude higher than those generated by scintillator screens in a fluoroscopy system [70]. The signal chain of FPD begins at the conversion layer, which follows either an indirect or direct design. In indirect-conversion detectors, a scintillator screen composed of Thallium-doped cesium iodide (CsI:Tl) absorbs incident X-rays and converts them into visible light photons. This structured CsI configuration utilizes needle-like crystals that act as light guides, directing photons toward an a-Si photodiode integrated into the individual detector element (dexel), where the optical signal is converted into proportional electrical charges. Conversely, direct-conversion detectors utilize a semiconductor layer, such as amorphous selenium (a-Se), to convert X-ray energy directly into electron-hole pairs under a high voltage bias, eliminating the stage involving intermediate light production [55]. Regardless of the conversion method, the generated charge is collected and stored in a local capacitor located within each individual dexel, which is paired with a TFT switch. To read the detector, gate lines trigger the TFTs one row at a time. This allows the accumulated charge to flow down parallel drain (or data) lines, where it is captured and processed by column-based charge amplifiers. These amplifiers boost the analog signal before it is passed to Analog-to-Digital Converters (ADCs) for digitization and final image formation. It is worth noting that while this architecture preserves spatial resolution, temporal artifacts can arise. The intrinsic lag of a-Si photodiodes [63] and the persistence of scintillation luminescence—where visible photon emission continues after X-ray excitation ceases—can result in image ghosting (afterglow) at high frame rates. The physical origin of this lag is attributed to the deep trapping and subsequent emission of electrons within the a-Si diodes, as well as the inherent afterglow of the CsI scintillator [71]. Consequently, significant residual signals can persist; Hoheisel et al. demonstrated that it can take up to 5 s for the residual signal to decay below the signal level of the image’s darkest regions [70]. This phenomenon may necessitate algorithmic correction to subtract the residual signal from the prior frame [72].

The transition to flat-panel detectors (FPD) offered distinct advantages over traditional image intensifier (II) systems, particularly regarding image fidelity and physical design. The inherent digital nature of FPDs allows for advanced post-processing—a significant upgrade over fluorescence screen-based systems—while providing the geometric accuracy required for precise image guidance. Unlike II systems, FPDs provide a completely distortion-free readout [68], a much wider dynamic range, no geometric distortion, and a wider field of view (FOV). They exhibit excellent image uniformity across the field of view by eliminating the “vignetting” and “veiling glare” inherent to II/TV chains. Indeed, FPDs have been shown to surpass II-based detectors for cone beam CT (CBCT) in terms of FOV, contrast, and resolution [73]. Physically, the FPD’s compact, thin-profile design significantly improves ergonomics and patient access compared to the bulky vacuum tube architecture of the II. In terms of performance, these detectors demonstrate high DQE, high frame rates, high dynamic range, small image lag (<1%), and excellent linearity [74]. Published DQE values for FPDs are typically reported in radiographic operation at exposures of the order of µGy per image. For CsI/a-Si indirect FPDs, Granfors reported that representative zero-frequency values reach DQE(0) ≈ 0.77 [75]. Measuring DQE under fluoroscopic conditions is complicated by temporal dynamics of the system; as a result, the DQE values typically quoted for an FPD describe its radiographic operation rather than its fluoroscopic operation. At fluoroscopic exposures, additive electronic readout noise becomes a non-negligible fraction of the per-frame quantum signal, but with adequate lag correction, the FPD retains most of its radiographic DQE: Granfors et al. measured DQE(0) ≈ 0.77 at 150 nGy/frame—essentially unchanged from radiographic conditions—and a reduction in less than 15% even at 5 nGy/frame and 0.5 cycles/mm. When the same FPD was compared with a state-of-the-art image intensifier/CCD chain using identical methodology, the FPD retained higher DQE at radiographic exposures but converged to approximately equivalent DQE at fluoroscopic exposures (~8 nGy/frame), so the FPD’s DQE advantage over a traditional image intensifier is preserved in radiography but largely closes at fluoroscopic levels [75]. To circumvent this, modern systems utilize pulsed fluoroscopy, which delivers radiation in high-intensity bursts. By increasing the dose-per-pulse while maintaining a low total exposure, the detector operates in a higher-signal regime that bridges the performance gap between fluoroscopy and radiography. Ultimately, this combination of digital architecture, high frame rates, and geometric accuracy makes FPD-based fluoroscopy an ideal tool for the rigors of tumor tracking. By 2014, the technology had matured sufficiently for integration into modern proton therapy systems [76], transitioning these advanced motion management strategies from the laboratory to the treatment room.

## 3. The Evolution of FGPT in Particle Therapy

The early usage of fluoroscopy in particle therapy can be traced back to the pioneering efforts at the Harvard Cyclotron Laboratory (HCL) in the 1970s, necessitated by the unique challenges of treating ocular melanoma. In an effort to expand the then-novel proton therapy to broader anatomic sites, Suit and colleagues proposed that the precision of the Bragg peak could offer a conservative alternative to enucleation (surgical removal of the eye) for patients with choroidal melanoma [77]. This concept was validated pre-clinically by Constable et al. [78], and the first human cases of choroidal malignant melanoma were treated by Gragoudas et al. at HCL shortly thereafter [2]. While previous successes with treating intracranial targets using protons relied on rigid skeletal fixation—where immobilization of the skull guaranteed target stability—the eye retains significant independent mobility, requiring active motion management. In the initial protocol, the patient was asked to maintain voluntary fixation while clinicians monitored eye position through a Closed-Circuit TV (CCTV) system; this method allowed the treatment to be paused by a clinician using a hand-switch if eye movement exceeding 0.5 mm was observed [2]. However, this method could not detect an unlikely but crucial source of motion: the head and the eye moving in opposite directions, which would cause tumor misalignment while leaving the visual landmarks on the TV monitor unchanged. This limitation was resolved with the integration of a fluoroscopy system [79,80]. In this improved system, tantalum rings sutured near the tumor served as radio–opaque surrogates, allowing for direct fluoroscopic monitoring of the tumor itself. The combination of patient gaze fixation, image-based surrogate tracking, and beam suspension on detected motion remains the operational template for ocular proton therapy at the dedicated 60–70 MeV beamlines in current clinical use [3]. At the CATANA Centre, for example, the patient fixes on a small red dot delineated by a laser light to establish the treatment gaze angle, while “during the proton therapy the patient’s eye is monitored by a video camera, the position of the pupil marked on the display so that the tiniest movements can be immediately detected and irradiation can be suspended” [4].

The real-time visualization of internal anatomy during radiation therapy was first demonstrated in photon treatment by Leong et al. [81]. Using a setup that coupled a fluorescent screen to a high-speed camera, they captured images at 30 frames per second to visualize the motion of the oral cavity and soft palate during nasopharyngeal treatments. Building on this foundation, Ohara et al. at the University of Tsukuba pioneered the first respiratory-gated photon irradiation for metastatic lung tumors in the late 1980s [82]. By the 1990s, the National Institute of Radiological Sciences (NIRS) in Japan had adapted these gated irradiation concepts for carbon-ion therapy [83]. Although this implementation utilized orthogonal fluoroscopy to monitor internal organ motion, the gating signal itself was not yet derived from the radiographic images. Instead, an infrared LED placed on the patient’s chest wall functioned as a respiratory surrogate, triggering the carbon-ion beam via a synchrotron RF-knockout extraction mechanism [84]. While particle therapy centers were establishing these first gated protocols, commercial photon therapy systems were simultaneously maturing to integrate automated motion monitoring into daily practice. Platforms such as CyberKnife (Accuray, Sunnyvale, CA, USA) [85] and ExacTrac (Brainlab, Munich, Germany) [86] adopted room-mounted orthogonal X-ray imagers to monitor target motion directly during delivery. This parallel development in the photon sector provided the first robust, commercial-grade evidence that internal target tracking could significantly improve the precision of external beam delivery. More recently, MR-Linac systems integrating diagnostic-quality MRI directly into the treatment unit have entered routine clinical use. The 0.35 T ViewRay MRIdian [87] and the 1.5 T Elekta Unity [88] both acquire continuous cine MRI during beam delivery, providing direct real-time soft-tissue visualization of the tumor with no ionizing imaging dose.

In particle therapy, gated treatments triggered by fluoroscopy have evolved along two complementary trajectories: marker-based tracking, which utilizes implanted fiducial markers as surrogate targets, and marker-less tracking, which localizes the tumor or anatomical structures directly from native image contrast. To date, only marker-based approaches have achieved commercial maturity and routine clinical deployment [76]. In contrast, marker-less tracking systems remain largely confined to institutional research environments. The following sections review the development and status of both methodologies, beginning with the history of the marker-based fluoroscopy gating system developed in Japan.

### 3.1. Marker-Based FGPT and Hitachi Real-Time Gated Particle Therapy (RGPT)

**Figure 2:**
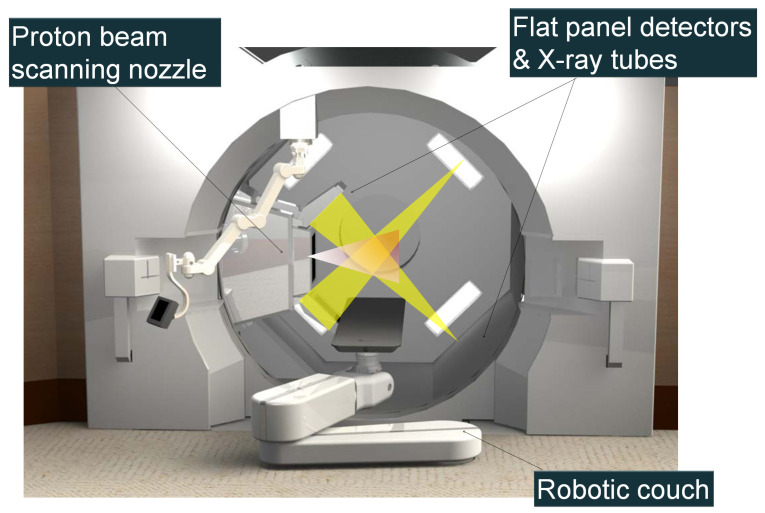
Hitachi RGPT Hitachi RGPT system on a rotating gantry. The horizontal orange beam represents the proton particle beam. The two mutually orthogonal yellow beams represent the on-treatment kV fluoroscopic imaging beams used for real-time fiducial-marker tracking; the corresponding kV X-ray tubes and flat-panel detectors are mounted directly on the gantry so that the imaging geometry rotates with the treatment beam. Reproduced from Shimizu et al. [76] under the terms of the Creative Commons Attribution License (CC BY 4.0).

The foremost commercial implementation of marker-based internal gating in particle therapy is the Real-time Gated Particle Therapy (RGPT) by Hitachi Ltd. (Tokyo, Japan) (Figure 2). This solution represents the direct evolution of extensive clinical research and technological innovation from a collaboration with Hokkaido University. The system adapts the pioneering tumor-tracking technologies called Real-Time Tumor-Tracking Radiotherapy (RTRT) originally established for conventional photon radiotherapy [89,90,91,92,93,94,95] to meet the rigorous demands of synchrotron-based proton delivery.

RTRT was the first implementation of fluoroscopy-triggered gated treatment. It was first implemented at Hokkaido University using a linear accelerator (Linac) in the late 1990s. In this system, patients were implanted with round gold fiducial markers of 2 mm diameter. During treatment, two sets of diagnostic fluoroscopes capture tumor motion in real-time. The locations of the fiducial markers were automatically processed using Otsu’s thresholding algorithm [96], and radiation was triggered when the gold marker was located in the planned position. The initial fluoroscopy imaging chain was based on image intensifiers and could take images every 0.03 s. The system achieved a tracking accuracy of 1.5 mm for tumors moving at speeds up to 40 mm/s [97]. To optimize the signal and minimize patient dose, the Linac pulse was synchronized to pause during X-ray acquisition, while the image intensifiers were activated only during the X-ray pulses [90]. This in-house tracking system also enabled researchers to perform detailed studies of tumor motion and hysteresis driven by respiratory and cardiac cycles [48].

The integration of real-time tracking was significantly aided by the evolution of beam delivery hardware. In passive scattering systems, the presence of large field-shaping devices at the nozzle often physically obstructed or narrowed the field of view of imaging devices. The transition to dedicated PBS systems removed these bulky accessories, creating the spatial clearance required to install fluoroscopy panels without compromising their imaging geometry or limiting their visibility of the target [98].

The successful implementation of respiratory-gated particle therapy requires first overcoming a layer of technical complexity greater than that found in photon therapy, primarily due to the specific constraints of the beam delivery mechanism. The challenge differs depending on accelerator architecture. In a cyclotron-based system, the magnetic field remains fixed and produces a continuous beam similar to a linear accelerator (Linac); as a result, the duty cycle and efficiency of gated irradiation are comparable to those of standard photon therapy. In contrast, a synchrotron system operates on a pulsed cycle, where the magnetic field must be ramped in synchronization with the increasing energy of the particles to maintain a stable circular trajectory. Beam extraction is restricted to the “flat top” phase of the magnet excitation pattern, making delivery efficiency highly sensitive to the synchronization between the synchrotron cycle and the patient’s respiratory phase. In a simulation study utilizing actual patient motion traces, Tsunashima et al. [99] found that a fixed magnet excitation cycle would increase average treatment times by a factor of three, suggesting that a variable magnet excitation pattern is essential for efficient gated irradiation. Addressing this, Hitachi Ltd., in collaboration with Hokkaido University, developed a “beam waiting” function that extends the flat top phase, enabling multiple gated irradiations within a single synchrotron cycle [100]. Using patient trajectory data collected from the RTRT system during the photon treatments, Matsuura et al. [101] determined the optimal gate window to be 2 mm for gated proton treatment. This series of investigations led to the clinical implementation of Real-time Gated Particle Therapy (RGPT) with Hitachi [76]. A hardware evolution in this system was the replacement of the image intensifier and optical coupling systems—standard in the previous photon-based RTRT system—with a modern FPD-based imager. This transition improved space efficiency, enabling the integration of orthogonal imaging units directly into the gantry alongside the spot-scanning nozzle. The system utilizes fluoroscopy to track implanted fiducials at 30 frames per second (FPS) with a tracking accuracy of 1 mm. The synchrotron operation cycle (injection, acceleration, waiting for the first gate, extraction, and deceleration) ranges from 2 to 7 s. While the beam waiting function improves efficiency by enabling multiple gates per cycle, the wait timer is limited to 200 ms to ensure stability [76]. The system latency—defined as the duration between the generation of the pulsed X-ray beam and the resultant proton beam-on/off—is set to 66 ms. Early clinical evaluations indicated that treatment time lengthening was manageable, ranging between 1.22 and 1.72 times the standard duration [76].

Two principal configurations of RGPT systems have been implemented clinically by Hitachi, each with distinct geometric and operational characteristics. In the first configuration, the X-ray source and flat-panel detector are mounted directly on the rotating treatment gantry, such that the fluoroscopic projection angle is inherently coupled to the treatment beam direction. In such cases, the treatment planning needs to carefully balance dosimetric optimality against the visibility of the fiducial markers on the fluoroscopy projection, as at certain gantry angles, overlying bony structures or other high-density anatomy may obscure fiducial markers, compromising tracking reliability. In the second configuration, the X-ray imaging system is fixed to the room infrastructure, with the X-ray tube installed on the ceiling and the detector panel mounted on the floor, resulting in fluoroscopic projections that remain geometrically constant irrespective of the treatment beam angle. Both configurations are being implemented at Mayo Clinic Florida, where the gantry-mounted system is deployed on the proton therapy gantry and the room-fixed configuration is installed at the carbon-ion fixed-beam port. For pulsed-fluoroscopy operation specifically, the Hitachi PROBEAT RGPT system operates at 70–125 kVp with tube currents up to 99 mA, and selectable pulse rates of 1, 7.5, 15, or 30 pulses per second chosen according to the target’s motion characteristics—typically 1 PPS for quasi-static targets such as prostate and 15–30 PPS for respiratory-driven liver, lung, and pancreatic lesions [102]. The pulse width is fixed at the installation level and is set within 2–3 ms. Beam quality of the X-ray imaging is specified by half-value-layer (HVL) acceptance testing, with a minimum permissible first HVL of ≥2.5 mm Al at 70 kVp rising to ≥5.4 mm Al at 150 kVp.

**Figure 3:**
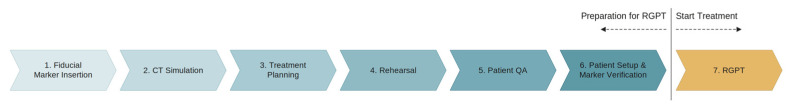
General RGPT clinical workflow.

**Figure 4:**
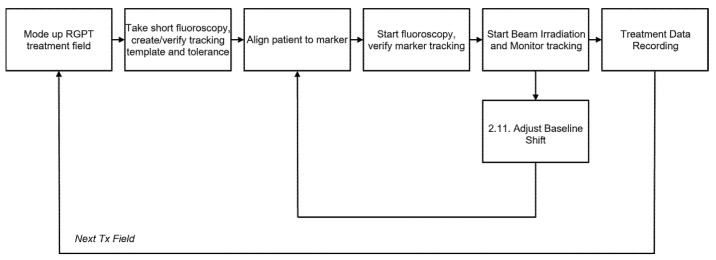
Typical RGPT workflow in a treatment.

Figure 3 illustrates the general RGPT clinical workflow, from fiducial marker insertion to treatment delivery. Figure 4 shows a typical RGPT field treatment workflow, detailing patient setup, template preparation, and gated treatment.

The dosimetric benefits of RGPT have been demonstrated in multiple simulation and validation studies. Shimizu et al. showed that RGPT dramatically improved dose conformity for hepatocellular carcinoma: while free-breathing spot-scanning achieved successful dose delivery (95–107% CTV coverage) in only 9/48 and 0/48 motion scenarios for two patients, RGPT achieved 48/48 and 42/48, respectively, while also reducing mean liver dose by approximately 50% for smaller tumors [76]. For lung tumors, simulation studies have demonstrated that RGPT with a ±2 mm gating width—i.e., the proton beam is permitted to remain on while the fiducial marker stays within a 2 mm radius of its planned 3D position—reduces dose error without substantially extending treatment time. [101]. Yamada et al. validated the reliability of RGPT dose delivery through log-data-based dose reconstruction across 168 fractions in eight liver cancer patients, confirming that delivered doses closely matched planned distributions [103]. Clinical outcomes are just beginning to emerge. Nishioka et al. reported the first prospective study of RGPT for prostate cancer, demonstrating 88.9% five-year biochemical relapse-free survival with early adverse event rates (8.9% ≥grade 2) that were non-inferior to conventional proton therapy [104]. While these results confirm the safety and feasibility of RGPT, comparative clinical outcome studies directly demonstrating superiority over non-gated approaches for moving tumors remain limited, highlighting an opportunity for future investigation.

Comprehensive commissioning and quality assurance protocols for RGPT have recently been published. Chen et al. described the commissioning of the Hitachi PROBEAT system at Johns Hopkins University, demonstrating that dose delivery to moving targets passed 3%/3 mm gamma analysis and that plan delivery uncertainty could be maintained within 2 mm [105]. Tan et al. reported the first published RGPT-specific commissioning and QA procedure, detailing six commissioning measurements including imaging quality, imaging dose, marker tracking accuracy, gating latency, tracking fidelity for irregularly shaped fiducial markers, and dosimetry. Their results showed gating latencies of 119.5 ms and 50.0 ms for beam-off and beam-on, respectively, with daily marker localization accuracy consistently below 0.2 mm [102]. Koh et al. presented the first comprehensive Failure Modes and Effects Analysis (FMEA) for RGPT following AAPM TG-100 guidelines, identifying 96 potential failure modes across the clinical workflow and highlighting irregular patient breathing as the highest-risk process [106]. Additional physics investigations have addressed treatment time efficiency, with Yoshimura et al. analyzing whether gated spot-scanning delivery can be completed within standard 30 min session times [107], and robustness evaluation methodology, with Lee et al. comparing algorithms for determining maximum allowable CTV shifts during RGPT for prostate cancer [108].

The imaging dose associated with continuous fluoroscopy during real-time tumor tracking needs to be considered in clinical implementation [109]. Shirato et al. reported that skin surface dose from a single fluoroscope in RTRT ranged from 29 to 1182 mGy/h depending on kVp and pulse width settings, concluding that precise dose estimation and reduction strategies are essential as lung RTRT treatment longer than 30 min of fluoroscopy can result in clinically significant cumulative imaging exposure [110,111]. Imaging dose can be reduced by using the lowest FPS setting suitable for the disease site, for example, the prostate often requires 1 FPS, whereas the lung may require 15 FPS. Postprocessing enhancement may further reduce the imaging exposure; for example, Miyamoto et al. showed that imaging dose can be reduced through motion-compensated image filters while maintaining tracking accuracy comparable to high-dose imaging [112].

The interaction between fluoroscopy and particle delivery creates a dual-challenge environment where each system can potentially compromise the accuracy of the other. While secondary radiation from the treatment beam can introduce noise into the imaging data, the inverse is also true: scattered fluoroscopic X-rays can infiltrate the dose monitor (DM) and be mistakenly recorded as proton monitor units (MU). Currently, the standard approach to mitigate this signal contamination is to momentarily pause during each fluoroscopy pulse to ensure that X-ray scatter is not active while the DM is recording, a method known as Interrupted Continuous Delivery (ICD). ICD reduces delivery efficiency, especially for particle beams delivered by Dose Driven Continuous Scanning (DDCS), intended for high-dose rate delivery. Recent investigation, however, suggests that ICD may be unnecessary for modern high-dose rate systems [113]. Yamanaka et al. found that a proton-beam current greater than 2 MU/s keeps target dose deviations within 1% of the planned distribution; the threshold for the organ at risk was 1 MU/s [113]. Regarding the converse effect where the particle beam affects the fluoroscopy imaging system, Terunuma et al. evaluated treatment beam-induced secondary-radiation background on the quality of fluoroscopy and found that 1.25% of the pixels are affected by sparse spikes and impact the mean-pixel-intensity variation remaining below 1% [114]. This may not affect visual perception, but it may affect the robustness of machine learning algorithms, which could be sensitive to out-of-distribution noises.

Although Hitachi’s PROBEAT/RGPT is the only commercially deployed system that performs continuous fluoroscopic intra-fraction tumor tracking in particle therapy, recent proton therapy vendors all provide substantial in-room kV imaging infrastructure (summarized in Table 1) on which comparable real-time tracking workflows could, in principle, be built.

Despite its clinical utility, marker-based tracking is subject to several limitations, ranging from complications associated with fiducial placement to significant dosimetric uncertainties. The most common complication is pneumothorax; Laurent et al. [118] reported incidence rates of 15% and 16.2% across two patient groups, while Geraghty et al. [119] observed a rate of 27% (226 out of 846 patients), and Collins et al. [120] reported rates as high as 30%. Pulmonary hemorrhage (hemoptysis) is another known risk. Furthermore, marker migration can compromise targeting precision [121]. The study by Kitamura et al. concluded that a planning target volume margin should be used to account for the registration uncertainty caused by marker migration [93]. The effect of fiducial marker migration was also studied by Shirato et al. [122], Imura et al. [123], and Van der Voort van Zyp et al. [124]. To mitigate marker migration, typically three non-collinear markers are used for 3D tracking [120], and Imura et al. suggest delaying tumor tracking radiotherapy until at least five days after insertion [125]. A rare but serious complication called tumor track seeding has also been documented by Patel et al. [126], who reported a case where a new tumor nodule developed around a gold fiducial, likely due to the needle dragging malignant cells through the track. However, they noted that while seeding may be common, implantation metastasis is rare because lung cancer cells generally have low growth potential in the pleural space. Finally, operational limitations also exist: not all patients are eligible for fiducial placement.

From a dosimetric perspective, high-density markers can cause Hounsfield Unit (HU) artifacts that introduce calculation uncertainties [13], which is particularly critical for particle therapy. Newhauser et al. found that a 2.5 mm tantalum marker used in proton therapy for uveal melanoma could create a dose shadow ranging from 22% to 80% [127]. Giebeler et al. demonstrated via Monte Carlo simulation that dose perturbation depends on marker size, orientation, and distance from the beam’s end of range [128]; they observed dose perturbations of 31% for large markers and 23% for medium markers in lateral opposed pair treatments. Habermehl et al. recommend utilizing only thin markers (<0.5 mm) or low-Z materials for hadron therapy to minimize these effects [129]. Matsuura et al. recommended utilizing 1.5 mm markers to avoid Tumor Control Probability (TCP) reduction [130]. Although they suggested that utilizing multiple-field irradiation could mitigate the underdosing effects caused by larger diameter markers, this could compromise the ability to spare surrounding critical organs.

Given the significant invasiveness and dosimetric uncertainties associated with fiducial markers, the ideal gating solution would be a non-invasive, marker-less tracking approach. Such methods aim to derive the gating signal directly from internal anatomical features, utilizing structures like the diaphragm as a surrogate or, most optimally, tracking the tumor mass itself to ensure precise beam delivery without the risks of implantation.

### 3.2. Marker-Less FGPT

**Figure 5:**
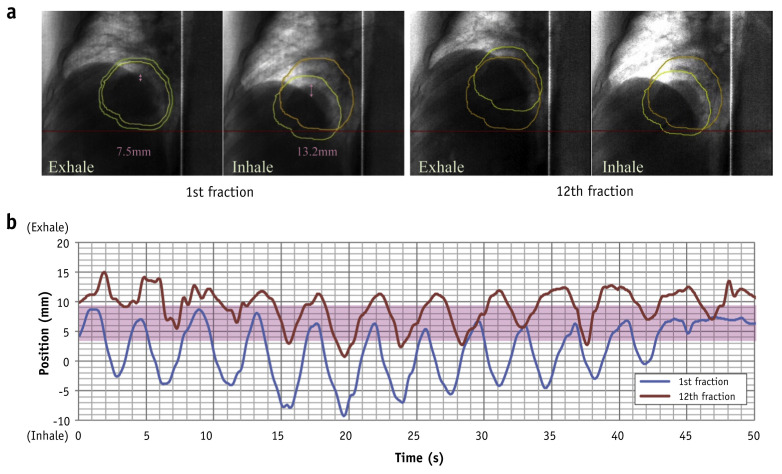
Marker-less tracking of lung tumor during carbon-ion therapy at NIRSreproduced from Reference [131] with permission. (**a**) Fluoroscopic images at exhale and inhale phases of the 1st and 12th treatment fractions. The green and orange contours delineate the PTV, and the yellow contour shows the CTV detected by the marker-less tracking algorithm; the carbon-ion beam is turned on whenever the tracked CTV lies inside the PTV; (**b**) Superior-inferior position of the tracked CTV as a function of time during the 1st (blue) and 12th (red) fractions. The pink shaded band marks the range of tracked CTV positions for which the carbon-ion beam is turned on.

Marker-less tumor tracking for particle therapy was pioneered at the National Institute of Radiological Sciences (NIRS) in Japan to support their carbon-ion PBS system [131,132,133]. NIRS launched a clinical trial in March 2015 with 10 patients (five lung and five liver), representing the first clinical application of marker-less tumor tracking for liver cancer in particle therapy. Treatments employed respiratory gating near end-expiration (typically phases T30–T70, but when motion within specific phase windows was deemed too rapid, T30 or T70 could be adaptively excluded to maintain stable gating). The initial implementation [131] reported an overall gating positional error less than 2.2 mm at the 95% confidence level, although the study used a non-traditional but clinically pragmatic definition of gating error—error was set to zero as long as the delivered CTV remained within the planned PTV. Under this definition, larger PTV margins can intrinsically yield smaller reported gating errors. Tracking accuracy was <2.5 mm, although the fraction of frames achieving a Tracking Registration Error (TRE) < 1 mm varied across patients (typically ~48–70%). The author showed that compared with simulated external gating (30% duty cycle based on external surrogates), the internal fluoroscopic approach produced substantially better gating accuracy, particularly for large-amplitude tumor motion. Figure 5 shows the marker-less tumor tracking system for carbon-ion pencil beam scanning treatment developed at NIRS, reproduced from Mori et al. [131] with permission.

The underlying tracking methodology integrates multiple-template-matching with machine learning augmentation to achieve robust target localization [134]. The multiple-template-matching framework relies on a library of “reference snapshots” constructed from pre-treatment fluoroscopy, where each template is explicitly associated with specific tumor coordinates and a respiratory phase [135,136]. During treatment, live fluoroscopic frames are compared against this library to identify the closest matches, hence infer the tumor’s position. To account for inter-fractional variations in breathing compared to the baseline sequence, the algorithm permits small translational shifts in the incoming image to maximize similarity. Rather than selecting a single best match, a voting strategy is employed to enhance robustness, aggregating tumor positions from all templates that exceed an empirical similarity threshold, typically between 85 and 95 training sets of approximately 60 images—roughly two breathing cycles—the multiple-template matching method achieved a reported tracking accuracy of ~3 mm. An interesting technical detail in the algorithm is that the respiratory phase is determined by the intensity of the fluoroscopic images: Berbeco et al. observed that fluoroscopic intensity fluctuates predictably with respiration, typically appearing darker during exhalation and brighter during inhalation, allowing for the definition of the phase via an extracted intensity waveform [137].

The workflow originally developed at NIRS comprises the following steps:

- 4D-DRR generation: Create digitally reconstructed radiographs (DRRs) for each respiratory phase intended for irradiation, with the CTV projected onto each DRR.
- DFPD acquisition and registration: Before treatment, dynamic flat-panel detector (DFPD) fluoroscopy video is acquired over several breathing cycles; register each DFPD image to the corresponding 4D-DRR using a 2D–2D registration algorithm.
- Manual verification: An oncologist and physicist review the projected CTV positions on DFPD images at each phase and adjust as needed to ensure ground-truth accuracy.
- Optimization: Automatically optimize the number of templates, similarity thresholds/scores, confidence metrics, and the machine learning dictionary to finalize tracking parameters.
- Tracking: During treatment, an instantaneous fluoroscopy image is analyzed by multiple-template matching to determine tumor location and trigger a gating signal accordingly.

A critical phase of this process involves manual verification, where an oncologist and physicist review the projected Clinical Target Volume (CTV) positions on DFPD images to ensure ground-truth accuracy before automatically optimizing tracking parameters. However, this “human-in-the-loop” requirement presents a significant operational bottleneck, as providing manual ground-truth tumor positions for training images can take up to 10 min per patient [131], thereby significantly limiting clinical throughput. Recent research at NIRS has increasingly focused on leveraging these neural networks to replace manual labeling and optimize model preparation [133,138,139,140,141,142,143]. Deep learning represents a transformative opportunity to overcome efficiency hurdles in marker-less tracking by automating feature extraction and ground-truth generation. By eliminating the need for manual curation, deep learning algorithms can streamline the preparation of template libraries and machine learning models. This advancement is particularly critical as therapy systems transition toward more efficient delivery modes where the concurrent operation of imaging and treatment beams demands highly robust, automated tracking to maintain dosimetric accuracy. The shift toward deep learning-based marker-less tracking could be the key to unleashing the full potential of marker-less FGPT. Table 2 provides a concise summary of the marker-based and marker-less FGPT systems discussed in the preceding text.

Marker-based and marker-less fluoroscopic tracking deliver different patient imaging doses on the same vendor system at identical kVp, mA, and pulse-width settings. The reason is geometric: marker-based tracking follows a small fiducial marker, so the minimum X-ray FOV is set by the fiducial’s motion amplitude alone. Marker-less tracking follows the soft-tissue tumor and typically requires anatomical context for the registration; the minimum FOV is therefore the geometric sum of the projected tumor diameter, the motion amplitude, and an algorithm-dependent context margin, and is systematically larger than the marker-based minimum. Because patient dose-area product scales with FOV area at fixed exposure parameters, the imaging-dose advantages reported for marker-based RGPT do not transfer one-to-one to marker-less FGPT.

## 4. Harnessing AI for Marker-Less Tumor Tracking: Opportunities and Challenges

As of May 2026, no commercially deployed FGPT system uses artificial intelligence for soft-tissue tumor localization during particle therapy. The only mature commercial FGPT—Hitachi’s PROBEAT RGPT performs fiducial localization by traditional template matching. The first and, to our knowledge, only clinical implementation of marker-less fluoroscopic tracking of soft tissue in particle therapy is the NIRS carbon-ion system pioneered by Mori and colleagues [131,132,133,134], which uses multiple-template matching with machine learning augmentation rather than an end-to-end deep neural network. The deep-learning algorithms surveyed in the remainder of this chapter, including the direct follow-ups to the NIRS work [139,140,141,142], are preclinical research-stage demonstrations. Accordingly, the present chapter reviews algorithms for marker-less tumor tracking in the broader context of fluoroscopic image registration. Many of these methods were originally developed for photon radiotherapy but are directly transferable to particle therapy, since the underlying kV fluoroscopic imaging chain is essentially identical across modalities.

### 4.1. Central Challenge for Marker-Less Tumor Tracking Using Projection Images

Fiducial marker tracking via kV imaging has demonstrated high accuracy across multiple clinical studies [144,145], even for lower-quality MV images [146]. This proven reliability has paved the way for the commercial implementation of RGPT [76]. However, the mandatory use of implanted fiducials imposes significant procedural burdens—including the risks associated with invasive insertion and marker migration—limiting the technique’s applicability to specific anatomical sites. Marker-less tumor tracking, which localizes the target directly from native anatomical contrast to trigger gating signals, represents the ultimate paradigm shift in FGPT. While this approach holds the potential to expand the benefits of motion management to a broader patient population and disease sites, it presents significant technical challenges. The central challenge of marker-less FGPT is to develop algorithms that achieve “human-level” robustness: the capacity to reliably identify and track target structures despite the vast anatomical and image-quality variability encountered in clinical practice. Despite the rapid development of deep learning research and numerous proposals, achieving this degree of generalized robustness remains the foremost unsolved challenge for FGPT.

Accurate tumor tracking during radiation therapy requires robust real-time 2D/3D image-registration algorithms capable of aligning target structures on the 2D projection image acquired during treatment. Medical image registration has been the subject of extensive methodological development, spanning traditional optimization-based approaches [147,148,149,150] to more recent deep learning methods. An effective registration algorithm must be able to focus selectively on structures of clinical interest while ignoring irrelevant features—a task that is intuitive for humans but remains a significant challenge for computers. This challenge is particularly acute for fluoroscopic projection images, where three-dimensional (3D) anatomy collapses into a single two-dimensional plane, creating complex patterns of tissue overlap. Although trained clinicians readily identify target anatomy within these projections, replicating this semantic understanding computationally has proven difficult.

Traditional registration algorithms search for the extremum of an image similarity metric [151]—such as Sum of Squared Differences (SSD), Normalized Cross-Correlation (NCC), or Mutual Information (MI)—as a function of registration parameters. While these metrics have achieved some success, significant limitations remain. The extremum of a similarity metric does not necessarily correspond to alignment of the clinically relevant structure, leading to erroneous registrations when the objects to be aligned are far apart or when irrelevant interfering features (e.g., bone projections over a lung tumor) dominate the image. This limitation is evident when positioning patients using orthogonal kV projection images at the treatment console. Automatic registration frequently fails if there is a large initial anatomic shift between the kV image and the reference digitally reconstructed radiograph (DRR). Currently, radiation therapists often need to perform manual pre-registration to bring landmarks into closer alignment before running the auto-registration. The problem is compounded when structures of interest are obscured by overlapping anatomy. Recently, deep learning has renewed hope for addressing these challenges by enabling algorithms to learn which structures to prioritize, though robust solutions remain an active area of research.

### 4.2. Emerging Deep Learning Algorithms

Over the past decade, deep learning has fundamentally transformed the research landscape of medical image analysis. The success of deep convolutional neural networks on natural image classification—achieving superhuman accuracy on certain benchmarks [152]—has inspired numerous efforts to develop systems that could assist or even replace humans in medical image analysis. Tremendous progress has been made across image-based diagnosis [153], image segmentation [154,155], image registration [156,157,158,159], and image reconstruction [160,161]. In radiation oncology, this is perhaps most evident in auto-segmentation, where deep learning models now delineate organs and tumor volumes with speed and precision that dramatically streamline clinical workflows.

The breakthrough success of AlexNet [162] on the 2012 ImageNet classification task revitalized the pursuit of human-level pattern recognition using deep neural networks in all aspects of industry, including the healthcare system. Deep neural network models are machine learning models parameterized by a large number of adjustable parameters that can be optimized (trained) using a large set of examples. A fundamental insight of deep learning, often referred to as the “scaling law”, is that the ability of deep learning models to generalize to new examples improves consistently as both the number of trainable parameters and the volume of training data increase. Notably, AlexNet, which brought revolution to the computer vision world, utilized an architecture structurally similar to LeNet [163] developed a decade earlier for written digit recognition, except that the number of parameters is orders of magnitude greater and the model was trained on a dataset order of magnitude larger. Three contributing factors drove the performance leap observed in AlexNet compared to the LeNet era of the late 1980s. The first factor is algorithmic efficiency: the calculus-based backpropagation algorithm accelerates parameter updates by at least a factor of 10 million compared to previous training methods. Secondly, hardware improvements over the intervening decades yielded a million-fold speed up in computation. Thirdly, efficient parametrization of the neural network using convolutional neural networks (CNNs) matches the inductive bias of natural images, reducing the number of parameters to be trained by a factor of millions compared to fully connected neural networks. These three factors multiplied, giving rise to the possibility of training very large models over very large datasets in the 2010s.

The historical trajectory of deep learning in medical image analysis closely mirrors developments in mainstream AI research, with methodologies and architectures originally designed for natural images frequently being transferred or adapted for medical applications. This parallel development is evident in the adoption of the VGG network [164]—a leading architecture in the 2014 ImageNet challenge—to achieve state-of-the-art performance in melanoma diagnosis [165] for the ISIC-2016 skin cancer classification challenge. Similarly, the Inception v3 architecture [166] developed in 2015 enabled dermatologist-level skin cancer classification [153]. The integration of ResNet [167] led to an architecture that secured the highest average classification performance across three skin cancer categories in the ISIC-2017 challenge [168]. In the domain of semantic segmentation, the fully convolutional network (FCN) introduced by Long et al. [169] established the encoder–decoder framework, which directly inspired U-Net [170]—the building block for numerous medical image segmentation models [154,155]. Generative Adversarial Networks (GANs) [171], introduced in 2014, experienced explosive adoption across a wide range of medical tasks, including disease classification, auto-segmentation, image registration, image reconstruction, and cross-modality image synthesis [172,173,174,175,176]. The attention mechanism [177], initially proposed for neural machine translation, achieved remarkable success in language modeling through the transformer architecture [178]. Its adaptation for computer vision, the Vision Transformer (ViT) [179], was rapidly integrated into medical image analysis as a potent alternative to traditional CNN-based architectures [180,181,182,183,184]. Most recently, Denoising Diffusion Probabilistic Models (DDPM) [185,186,187] have surpassed GANs in popularity for image generation tasks, quickly becoming a focal point in medical image related research [188,189,190,191].

There has been tremendous progress in applying deep learning to medical image registration [156,157,158,159]. Deep learning models for image registration typically fall into the following frameworks: methods based on direct parameter regression, methods based on segmentation, methods based on image synthesis, and others. Table 3 summarizes the algorithms reviewed in Section 4.2.

**Methods based on direct parameter regression**: Direct parameter regression constitutes one of the earliest paradigms in AI-based image registration, wherein neural networks predict the coordinates of the tumor or its bounding box. Neural network-based region proposal [217] and its variants [218,219] have been applied to pancreas [220,221], lung [222], and fiducial markers [223]. For this type of network, the models are trained to minimize a combination of regression loss and classification loss. CNN-based regression was also used to produce the Deformation Vector Field (DVF) [140,224,225]. The introduction of the Spatial Transformer Network (STN) by Jaderberg et al. [226] provided a popular differentiable module capable of explicitly warping an image within a neural network architecture. This mechanism has been widely adopted in medical physics for image registration. De Vos et al. utilized STNs for affine and deformable registration [192,193], while Li et al. applied the framework to non-rigid registration tasks [194]. The VoxelMorph model [195,196] integrated STN-based warping to enable rapid learning of deformable registration fields. A variation called CycleMorph [197] incorporated cycle-consistency constraints into this architecture to improve topological preservation. With the advent of diffusion model, DVF generated by a diffusion model has also been proposed [188].

**Methods based on segmentation**: Segmentation-based methods have emerged as a popular paradigm due to the success of U-Net [170] for pixel-level delineation of targets or surrogates [155]. The idea of this approach is to segment the structure of interest from the projection images and then use the segmentation for image registration. This has been applied to track fiducial markers [144], spine [198], pancreatic stent [199], as well as soft tissue such as diaphragm [200] or tumor itself [139,141,201,202,203,204]. A marker-less lung tumor tracking algorithm was developed by Hirai et al. [139] at NIRS in order to improve upon their existing multiple-template matching algorithm. In their deep learning model, a tumor probability map (TPM) was predicted from a structure similar to a U-Net. The trained model has an inference time of less than 40 ms, thus available for real-time tracking at 15 FPS. Tracking accuracy was tested for 10 patients (five lung and five liver) and found to have an average tracking error less than 2 mm. The drawback, as the authors pointed out, is that it was trained with treatment-planning 4DCT data hence may not be able to capture changes between simulation and treatment [139].

**Methods based on image synthesis**: Image synthesis became popular with the success of GAN [171]. The remarkable ability of GAN for super-resolution [227] or style transfer [228] fits naturally with the wish of medical physicists to improve image quality or to match images from different modalities. For this reason, GAN became the indispensable component for numerous models [172,173,174,175,176]. In the context of image registration on a 2D projection domain, techniques now exist to synthesize volumetric information in less than 1 s from single projection images to assist with registration [205]. ResNet-based GANs have been employed to decompose kV images for enhanced registration for spine SBRT [206]. Fu et al. [207] built a patient-specific model to convert on-treatment KV projections into synthetic DRR images with enhanced tumor visibility for downstream on-treatment tumor tracking [208]. DRR generated from pre-treatment CBCT was also proposed to improve marker-less tracking for pancreas SBRT [209]. In the context of tracking the pancreas, Ahmed et al. published a contour prediction model based on patient-specific fine-tuning of a GAN-based population model [229]. The model was able to achieve a 95-percentile Hausdorff Distance (HD95) within 5 mm for 90% of the tests, and the inference speed is about 30 ms. Tracking based on prostate segmentation on KV projection was proposed by Mylonas et al. [204]. Yan et al. published tracking from fluoroscopy images from a color image intensifier using synthetic DRR generation [211,212].

**Other methods:** Besides the above three dominant approaches, the Recurrent Neural Network (RNN) has been used to predict tumor motion from transponder signals [213]. Siamese networks have introduced robust patient-specific similarity learning [214]. More recently, vision transformers have been incorporated for affine registration [215]. A Zero-shot learning framework proposed by Xu et al. combines a traditional image similarity measure with a pre-trained deep neural network for template matching [216]. Their method also provides an uncertain measure for the prediction.

### 4.3. Challenges of the Current Deep Learning Algorithm

Although there has been tremendous progress in applying deep learning to medical image registration [156,157,158], the application of these powerful tools to medicine carries uniquely high stakes. Unlike natural image classification, where an error amounts to a mislabeled photograph, radiation therapy is a mission-critical domain in which mistakes translate directly to patient harm—underdosed tumors or overdosed organs at risk. This demands a standard of reliability far exceeding conventional performance benchmarks.

Deep learning approach in the present form has both advantages and disadvantages. One advantage of the deep learning solution is that the algorithm can be adapted to incorporate domain knowledge through training examples, unlike conventional algorithms, which use all-purpose image similarity metrics not specialized for the underlying tasks. In addition, deep learning models allow registration parameters to be directly predicted through a feed-forward neural network, resulting in an algorithm superior in speed compared to traditional methods that involve iterative optimization. A disadvantage of deep learning, however, is that a feed-forward neural network is opaque in what features are used and provides no explanations or uncertainty measure of its prediction. This black box approach creates a trust barrier that hinders the wide adoption of the technology. Conventional algorithms, on the other hand, are more transparent in this aspect, as the numerical value of the similarity metric provides its “explanation” for the prediction.

At the heart of the deep learning approach is the notion of generalizability [230]. The hope is that a model trained on a finite dataset can perform sufficiently well on unseen data. Because medical and natural images vary greatly at the pixel level—due to differences in viewpoint, exposure, detector noise, and artifacts—a model that generalizes well must capture high-level abstractions while remaining robust to irrelevant low-level variations. Numerous deep learning models have been proposed for image registration [156,157,158], but their robustness has not been thoroughly studied. Some intriguing aspects about deep learning raise concerns about the intelligence of these algorithms.

**Adversarial Examples**: It is a surprising failure mode where insignificant noises can cause a well-trained deep learning model to fail [231,232]. Adversarial Examples raise questions about whether deep learning algorithms have gained the required “intelligence” to perform mission-critical tasks such as image registration for stereotactic body radiation therapy (SBRT). Given the fact that deep neural networks have achieved superhuman performance on image classification tasks [152], it is tempting to assume that these models have acquired perceptual features analogous to those used by humans. Thus, it came as a surprise when adversarial examples were first discovered [231]. The authors found that input altered by negligible noises can cause a machine learning model to fail on tasks that were initially performed correctly. These can be considered as optical illusions for an AI system. Often, such attacking noises are insignificant and imperceptible to human observers. Nevertheless, it has been shown that adversarial examples exist in all popular modern deep learning vision systems. In a recent study, Jo et al. discovered that convolutional neural networks tend to learn superficial statistical clues rather than high-level abstracts [233]. In their experiment, a well-trained convolutional neural network generalizes poorly to natural images smoothed by a Fourier filter, losing up to 28% of its generalization accuracy. In another study, Azulay et al. found that a one-pixel shift or one-pixel rescaling of an image can result in a dramatic change in a network’s output [234]. Adversarial examples are also present in diagnostic AI and image segmentation models [235,236,237,238,239]. For example, Ozbulak et al. showed that a genuine breast image, initially classified as 95% cancerous by a deep learning model, can be perturbed by small adversarial noises, resulting in a prediction at the other extreme—classifying the tissue as healthy with 99% confidence [236]. In an experiment on brain tumor segmentation over MRI images, Cheng et al. found scenarios where adversarial examples cause significant drops in contouring accuracy for a UNet-based deep neural network [237].

**Hallucination**: Another aspect that raises concern about the robustness of AI-based algorithms is the phenomenon called hallucination. AI hallucination refers to the phenomenon where artificial intelligence systems generate outputs that appear plausible and confident but are factually incorrect, fabricated, or unsupported by the input data or reality. In large language models (LLMs), hallucinations manifest as fluent, coherent text containing false information, fabricated citations, or statements that contradict established facts—often delivered with unwarranted confidence [240,241]. In medical imaging and computer vision, hallucinations are well documented and present as false structures, phantom lesions, or anatomical features that appear visually realistic but do not exist in the ground truth, potentially compromising diagnostic accuracy [242,243,244,245,246]. The underlying causes of hallucinations are not completely understood: they may arise from biased or insufficient training data, the intrinsic probabilistic nature of deep learning models, limited understanding of context or visual features, or the model’s tendency to extrapolate beyond its training distribution. Regardless of the application domain, hallucinations share a common characteristic—they produce outputs that are plausible but incorrect, posing significant risks in high-stakes applications such as healthcare, legal, and scientific contexts where accuracy is paramount.

**Peculiar Data Augmentation Requirement**: Analyzing the way deep neural networks for image registration were trained also reveals some intriguing aspects that do not represent the robustness of human intelligence. For example, for regression-based registration models, the networks are typically trained with a pair of fixed and moving images and asked to produce the registration parameters (translations, rotations, etc.) that would bring the fixed and moving images into alignment. For such a feed-forward network, one finds that the model trained using pairs of input images with relative shifts up to 10 pixels will often make large test-time errors on input images with larger shifts, e.g., 15 pixels. The lack of geometric generalizability raises questions as to whether this type of network has learned to identify relevant high-level features to perform reliable registrations. Currently, this type of neural network needs to be trained on a dataset augmented significantly to cover all possible geometric transformations, leading to a training set that grows polynomially with the range of registration parameters. For instance, for a 3D registration network that predicts six parameters (three shifts and three angles), doubling the range of prediction requires a 64-fold augmentation of the training set. To avoid overfitting, the neural network model must be made larger (in terms of the number of parameters). Hence, the model must be trained for a much longer time, all to cover trivial geometric transformations that seem unnecessary to humans.

**Lack of Geometric Stability**: Testing whether registration error remains independent of initial misalignment represents one of the benchmarks for evaluating both the intelligence and the robustness of a registration algorithm. An algorithm exhibiting true translation-invariant behavior would demonstrate consistent accuracy regardless of the magnitude of initial misalignment—mirroring the human capacity for correspondence identification across arbitrary displacements. To date, however, no method has consistently demonstrated this capability. Most deep learning-based algorithms exhibit increasing registration error as initial misalignment grows, a pattern distressingly similar to the behavior of classical optimization-based approaches. This vulnerability suggests that despite architectural advances, current methods have not fundamentally solved the geometric robustness problem inherent to registration in the projection domain. Achieving reliable, initial-alignment-independent performance remains an open question facing 2D/3D image registration on the projection domain.

The peculiarities of the aforementioned aspects in current AI-based algorithms, and the fact that despite numerous marker-less tracking models published in the literature, robust real-world clinical implementations remain rare, underscore the inherent challenges of current deep learning approaches. Robust medical image analysis is intrinsically predicated on a 3D understanding of the physical world. This is particularly evident in the interpretation of 2D projection images; human experts navigate this data by leveraging an implicit 3D mental model, which provides the cognitive basis for anatomically decoupling overlapping tissues even when they appear superimposed in the 2D plane. While the prevailing success of artificial intelligence in the industry is currently exemplified by large language models (LLMs), their underlying methodology does not translate seamlessly to the visual domain. LeCun has emphasized that the dimensionality and variance of image pixel space are astronomical compared to those of discrete language tokens [247]. Whereas LLMs perform stochastic prediction over a finite vocabulary—effectively navigating a massive probability table—the infinite variations in pixel intensities render such a “next-pixel” prediction approach computationally and theoretically intractable for computer vision. The pursuit of “true intelligence” in medical imaging hinges on the underlying representation of the images. Balestriero et al. showed that learning paradigms prioritizing pixel-level reconstruction accuracy may be counterproductive, as the pursuit of superficial fidelity can degrade the underlying semantic representations learned by the model [248]. From this perspective, a GAN, ViT, or DDPM-based model developed with a loss function modified to improve pixel-level precision for the reconstructed images would be fundamentally limited in capturing the true representation of the 3D model. To capture the authentic variance of the physical world, LeCun advocates for a fundamental architectural shift toward world models—systems that perform prediction in latent space rather than raw pixel space [249,250,251]. Similarly, Li maintains that a foundational breakthrough yet to happen in AI is sophisticated 3D reasoning [252]. Without these advancements, deep learning models remain limited in their capacity to internalize the complex, 3D physical constraints requisite for high-stakes medical interventions.

### 4.4. Emerging Directions

Several directions are emerging in the field of AI and may lead to addressing the limitations of conventional deep learning in medical image registration, offering potential pathways toward more robust and clinically deployable solutions.

#### 4.4.1. Patient-Specific Models

The inherent difficulty in training a universal model capable of generalizing across an entire patient population is evidenced by the proliferation of patient-specific models instead for motion management in the recent literature [141,202,203,207,209,210,253,254]. Given the nearly infinite variations in image morphology—including tumor size, texture, and shape—a population-level model may exceed the capacity of current deep learning architectures [247]. Consequently, patient-specific approaches are favored, as they operate under a significantly reduced learning burden by constraining the domain of visual variance to an individual’s unique anatomy. However, critical concerns persist regarding the clinical implementation of these models, particularly concerning temporal generalizability and anatomical drift; if a model is trained exclusively on planning CT images, it may fail to generalize to inter-fraction anatomical changes occurring by the day of treatment. This lack of generalizability to “on-the-day” anatomy remains a pervasive hurdle for robust motion tracking. To mitigate these discrepancies, the ideal approach would be training a treatment fraction-specific model based on images acquired immediately prior to delivery (e.g., daily CBCT). The primary barrier of this approach is the high computational cost of training. Current deep learning models typically require hours or days of GPU processing—resources often limited in a clinical hospital environment, making the training of sophisticated patient-specific registration models within minutes a major technical challenge. This necessitates the development of entirely new, rapidly adaptable model architectures.

In a runtime-trained system, the model weights used to treat a given patient are generated minutes before beam-on and are unique to that patient. Therefore, they cannot be pre-approved as a fixed algorithm under the current Pharmaceuticals and Medical Devices Agency (PMDA) or Food and Drug Administration (FDA) frameworks. We expect that clinical translation will require a regulatory model analogous to treatment-planning QA in radiation oncology: the training procedure and its verification harness are cleared once, but each patient’s model undergoes a per-patient acceptance test signed off by a qualified medical physicist before treatment. This acceptance test should produce human-judgeable artifacts, such as a predicted-versus-actual tumor overlay on held-out fluoroscopy, explainability maps, and uncertainty estimations. Responsibility for the per-patient model would then be shared among the device manufacturer, the institution, and the certifying physicist. A detailed regulatory framework for this paradigm will need to be developed collaboratively before routine clinical use.

#### 4.4.2. Incorporating Explainability

For AI to gain trust from human users, it must be able to explain the underlying reason for its prediction. This provides a mechanism for clinicians to assess whether a model’s decision aligns with sound clinical reasoning and to identify cases where the algorithm may be relying on spurious correlations rather than meaningful features. Explainability has become a central topic of discussion as AI algorithms are increasingly adopted in daily life and high-stakes decision-making [255,256,257,258]. Regulatory frameworks, including the European Union’s General Data Protection Regulation (GDPR) and AI Act, have codified a “right to explanation” [255] for automated decisions that significantly affect individual—underscoring that transparency is not merely a technical desideratum but a legal and ethical imperative. Equally important is the capacity of an AI algorithm to provide a confidence level or uncertainty measure, particularly for deployment in healthcare settings where erroneous predictions carry significant harm [259,260,261,262]. The current incarnation of deep learning architectures, however, has overwhelmingly emphasized point-estimate prediction accuracy while neglecting uncertainty quantification. Most feed-forward neural networks produce deterministic outputs without any indication of whether a given prediction lies within the model’s domain of competence or represents extrapolation into unfamiliar territory. This omission is problematic: a model may output a confident-appearing prediction even when the input deviates substantially from the training distribution, providing no warning to the end user. In clinical practice, such silent failures can be catastrophic. A robust AI system should not only provide accurate predictions but also “know what it does not know”—flagging cases of high epistemic uncertainty for human review rather than presenting all outputs with false equivalence. Achieving this dual capacity for accurate prediction and reliable uncertainty estimation represents a prerequisite for the trustworthy integration of AI into real-world clinical workflows.

Several efforts have been made to incorporate uncertainty estimation into medical image-registration frameworks. Within the context of traditional statistical learning, Risholm et al. introduced a non-rigid Bayesian registration framework that estimates the posterior distribution on both deformation and elastic parameters, enabling visualization of registration uncertainty for neurosurgical guidance [263]. Le Folgoc et al. investigated uncertainty quantification under a sparse Bayesian model, implementing reversible jump Markov Chain Monte Carlo sampling to characterize the posterior distribution of transformations [264]. With the advent of deep learning, Dalca et al. developed VoxelMorph-diff, a probabilistic diffeomorphic registration framework built on convolutional neural networks capable of providing uncertainty estimates alongside registration outputs [265]. Khawaled et al. proposed NPBDREG, a fully non-parametric Bayesian framework employing stochastic gradient Langevin dynamics to characterize the posterior distribution without restrictive Gaussian assumptions [266]. Gong et al. introduced an uncertainty learning approach for unsupervised deformable registration that predicts registration uncertainty guided by reconstruction error [267]. More recently, Rivetti et al. developed a CNN-based model capable of jointly performing deformable image registration while predicting its associated uncertainty [268], and Xu et al. proposed models that incorporate learned similarity measures with uncertainty prediction [216]. Despite these advances, the capacity to provide reliable uncertainty estimates remains underdeveloped across most registration algorithms. A continued emphasis on equipping models with principled uncertainty quantification is essential, not only for enabling rigorous robustness evaluation but also for fostering the clinical trust and regulatory acceptance necessary for widespread adoption in mission-critical applications such as image-guided radiation therapy.

Beyond its role in building clinical trust, explainability can serve as a concrete enabler of regulatory approval for AI-driven FGPT systems. Saliency and attention maps allow regulators to verify that a deep learning marker-less tracker attends to anatomically meaningful structures such as the diaphragm contour and the tumor mass—rather than spurious correlations, thereby strengthening the equivalence argument when existing template-matching systems serve as the predicate device. Concept architectures, in which predictions are routed through human-interpretable intermediate variables such as diaphragm position or tumor centroid, enable performance specifications to be written in clinically meaningful terms rather than as opaque input–output behavior. Counterfactual analyses identifying the minimum input change that would alter a gating decision support systematic hazard identification by directly probing the decision boundary, connecting naturally to the adversarial robustness concerns discussed above. Uncertainty quantification provides the per-prediction confidence signal needed to operationalize selective-prediction safety nets: the system can abstain from autonomous gating and defer to the operator when confidence falls below a validated threshold. Together, these XAI techniques transform explainability from a desirable property into a practical toolkit for navigating the regulatory pathway toward clinically deployable marker-less FGPT.

A fully specified stress-test protocol for AI-based FGPT is beyond the scope of this review and will require a dedicated standardization effort. We propose, however, that any such protocol should encompass at least the following four categories of testing. (i) Geometric robustness testing, in which the model is challenged with controlled perturbations of the input geometry—initial-position offsets, rotations, scaling, and image translations—to characterize how tracking accuracy degrades with departures from the planning-time baseline. (ii) Image-quality robustness testing, in which the model is challenged with reduced fluoroscopic exposure (lower mAs and pulse width), increased Poisson and electronic noise, scatter and beam-hardening artifacts, anatomical occluders such as implanted hardware, and acquisition characteristics representative of vendors and institutions not seen during training, in order to characterize generalization beyond the training distribution. (iii) Adversarial robustness testing for the deep neural network itself, including both worst-case adversarial perturbations bounded and clinically realistic distractors such as decoy high-contrast structures (ribs, spinal hardware, surgical clips). The goal is to quantify the gap between in-distribution accuracy and behavior under inputs that are visually plausible but pathologically constructed. (iv) Explainability testing, in which post hoc explanations of the model’s predictions (e.g., saliency or attribution maps) are evaluated for consistency under repeated and slightly perturbed inputs, for fidelity to the model’s actual decision pathway, and for plausibility against clinician-annotated regions of interest, to guard against models that achieve apparent accuracy by relying on anatomically irrelevant features. We anticipate that future community standards will define quantitative pass thresholds for each category.

#### 4.4.3. Emerging Deep Learning Paradigm

While patient-specific models may offer a pragmatic solution to immediate clinical needs, real change needs to come from a more intelligent model that is able to capture the underlying representation. Some new models occurred in the industry serve as the next candidates for medical physicists to develop more robust algorithms.

**Geometric Deep Learning**: The historic success of convolutional neural networks (CNNs) is largely attributable to their built-in translational equivariance, where shifting an input causes a corresponding shift in feature maps, enabling efficient weight sharing [269]. However, standard CNNs are inherently limited by their inability to respect more complex geometric structures, such as rotations, scalings, or the non-Euclidean curved geometry of anatomical surfaces. Geometric deep learning addresses these limitations by extending neural architectures to manifolds, graphs, and point clouds, encoding arbitrary symmetries directly into the network design [269,270,271,272]. Group equivariant neural networks, for instance, guarantee that outputs transform predictably under various geometric operations, which may help to build a principled framework for image registration that respects geometric constraints. Suliman et al. applied geometric deep learning to surface registration [273]. Greer et al. published a registration framework based on geometric deep learning principles with a coordinate attention mechanism [274]. Graph Neural Networks (GNNs) represent a parallel advancement in this domain by treating anatomical structures as nodes within a connected graph, allowing for the explicit modeling of spatial relationships and biomechanical constraints. This was exemplified by the work of Shao et al., who utilized GNNs for real-time liver tumor localization from sparse X-ray projections [275]. By performing node-based perceptual feature pooling on a liver surface mesh, their model predicts boundary DVFs, which then drive a biomechanical model to estimate internal tumor motion. The model is applied to liver motion estimation.

**World Models**: Beyond specialized geometric priors, a broader paradigm shift is occurring through the development of world models designed to learn general-purpose representations of physical reality rather than task-specific mapping functions. In the context of 3D understanding, Neural Radiance Fields (NeRF) have introduced continuous volumetric functions capable of synthesizing novel views from sparse observations [276]. This move toward “spatial intelligence” is being spearheaded by researchers at the World Labs, aiming to build large-scale world models capable of perceiving and reasoning about 3D environments. Their Marble platform, which generates spatially consistent 3D worlds from 2D inputs, suggests a future where medical simulations are driven by models that possess an inherent understanding of 3D space. Such architecture offers a path toward medical AI that can conceptualize the complex, 3D physical objects in the human body. Adaptations of NeRF have been used in medical image synthesis. MedNeRF has shown that these continuous representations can reconstruct CT-like volumes from limited X-ray projections by disentangling surface shape from internal depth [277]. On parallel development, Gaussian splatting has emerged as a high-speed alternative that represents scenes via explicit 3D Gaussian primitives, enabling rapid sparse-view CT reconstruction and multimodal surface modeling [278,279,280]. Another approach toward world models, advocated by LeCun, is the Joint Embedding Predictive Architecture (JEPA) that provides a foundational framework for autonomous machine intelligence that learns world models through passive observation [247]. Unlike generative models that struggle with the high dimensionality of raw pixels, JEPA operates in a restricted representation space, predicting the “latent state” of missing or future observations. Implementations such as I-JEPA [249] and V-JEPA [250,281] have demonstrated that this latent-space reasoning allows models to focus on semantically meaningful features while ignoring irrelevant noise [249]. By pre-training on massive video datasets, these models achieve state-of-the-art motion understanding and can even transfer learned representations to zero-shot active control tasks [250].

#### 4.4.4. Inference Time Optimization Using Energy-Based Model (EBM)

Most contemporary deep learning approaches for medical image registration rely on feed-forward neural networks to directly regress displacement fields or registration parameters in a single, constant-time pass. However, these static architectures are often ill-suited for the high-dimensional variability of clinical imagery. EBMs represent a framework designed as a strategy to bypass the limitations of traditional statistical learning [282]. While standard machine learning is often constrained by the requirement to model normalized probability distributions—a task that some argued to have “handicapped” machine learning—the EBM approach discards probabilistic normalization. Instead, the model learns a scalar energy function trained to assign minimum energy values to predictions that are compatible with observed reality and higher energy to those that are not. A critical advantage of EBMs is their capacity for inference-time optimization. Unlike traditional non-EBMs that produce outputs via a fixed computational budget, EBMs iteratively search for the energy minimum. This process mirrors human expert behavior in image registration; a radiologist or medical physicist does not take a constant time to align complex images. Instead, they devote a variable, indefinite amount of time to “reasoning” through difficult cases where anatomy is significantly deformed or occluded. This mechanism aligns with the cognitive transition from “System 1” (fast, intuitive) to “System 2” (slow, deliberate) thinking [283]. By shifting from rigid, one-pass regression to an iterative energy-minimization paradigm, EBMs may allow the model to handle wider variations and anomalies. This flexibility ensures that the output is not just a statistical guess but an optimized solution, ultimately bringing significantly more robustness to real-time clinical workflows.

#### 4.4.5. Hardware Improvements

Beyond algorithmic advances, hardware innovations offer complementary pathways to improved tumor localization. Dual-energy (DE) imaging exploits the energy-dependent attenuation of X-rays to decompose images into tissue-specific components, enabling bone suppression that dramatically improves soft-tissue tumor visibility in fluoroscopic tracking. Dhont et al. provided foundational work on dual-energy CT applications in radiotherapy, demonstrating improved electron density estimation and tissue characterization for dose calculation accuracy [284]. Menten et al. demonstrated that dual-energy imaging could enhance automated lung tumor tracking for real-time adaptive radiotherapy by generating radiographs with reduced bone visibility, improving tracking success from 90.7% in single-energy images to 99.9% in dual-energy frames [285]. More recently, Haytmyradov et al. developed a benchtop fast-kV switching dual-energy fluoroscopy system for marker-less tumor tracking, demonstrating the feasibility of real-time DE imaging on clinical linear accelerators with on-board imaging systems [286]. These hardware-based approaches circumvent many limitations of software-only bone suppression algorithms by directly acquiring the physics-based information needed for tissue decomposition, though they require specialized imaging equipment and careful optimization of energy pairs and weighting factors.

Imaging hardware is only one half of the hardware story for real-time marker-less AI-based FGPT; the other half is the computing hardware that runs the deep network on every fluoroscopic frame within the system’s hard latency budget. AAPM Task Group 264 defines real-time system latency in radiotherapy as ≤500 ms [287], and the deployed Hitachi RGPT precedent achieves a total system latency of approximately 100 ms [76,102,105]. At the 15–30 fps frame rates typical of FGPT fluoroscopy, the per-frame interval is 33–67 ms; after image acquisition, transmission, and beam-control signaling are accounted for, the budget remaining for the deep-network forward pass is on the order of 200–300 ms. Closing this gap requires GPU and AI-accelerator hardware deployed at the gantry and inference-runtime optimization. A separate compute concern arises in the patient-specific paradigm proposed in Section 4.4.1: per-patient model training must compress from the hours-to-days typical of research workflows to minutes that fit between simulation and treatment, which adds a high-throughput on-site training infrastructure to the clinical equipment list. Computing is, therefore, not a secondary engineering detail but a primary determinant of which algorithmic ideas will reach a treatment vault.

## 5. Conclusions

Fluoroscopy-guided motion management is essential for realizing the full dosimetric advantages of particle therapy. This review has traced the evolution from image intensifiers to flat-panel detectors, which have enabled FGPT solutions such as Hitachi’s RGPT. Marker-based tracking has demonstrated clinical feasibility, but the invasiveness of fiducial implantation, associated complications, and dosimetric perturbations limit broader adoption. Marker-less tracking—directly localizing tumors from native anatomical contrast—remains the ideal but technically challenging solution due to the superposition of 3D anatomy onto two-dimensional projections. Deep learning offers the most promising path forward, with numerous architectures proposed for tumor localization in projection images. However, robust clinical translation remains elusive due to concerns about generalizability, adversarial susceptibility, and potential for hallucinated outputs—failure modes unacceptable in radiation therapy.

The path toward reliable, clinically deployable marker-less FGPT will likely require a convergence of advances: continued refinement of deep learning architectures with built-in geometric and physical constraints, hardware innovations such as dual-energy imaging for improved soft-tissue contrast, rigorous validation frameworks that test robustness across the full spectrum of clinical variability, and regulatory pathways that address the unique challenges of AI-driven real-time treatment adaptation. As particle therapy continues to expand globally, solving the marker-less tracking challenge will be essential to unlocking the full therapeutic potential of this technology for patients with thoracic and abdominal malignancies.

## Figures and Tables

**Table 1:** In-room kV imaging configurations of recent proton therapy systems.

Vendor/System	Gantry Rotation	kV Imaging Configuration	Volumetric Imaging	Reference
Hitachi PROBEAT (RGPT)	360°	(A) Gantry-mounted X-ray tubes + flat panels; (B) Room-fixed ceiling-mounted tubes + floor detectors	kV-CBCT and stereoscopic kV-kV planar	[76]
Varian ProBeam 360°	360° (±190°)	Two orthogonal kV imaging chains integrated into a gantry; 40–140 kV, 0.4–1000 mAs	Gantry-mounted kV-CBCT (80–140 kV) with full or half rotation	[115]
IBA Proteus PLUS/ONE	220°	Room-fixed kV-kV stereoscopic pair (two floor tubes 60° apart) + retractable gantry-mounted kV tube + detector	Gantry-mounted kV-CBCT	[116]
Mevion S250i HYPERSCAN	360°	Room-fixed: two orthogonal a-Si flat panels on ceiling rails	Separate medPhoton ImagingRing CBCT (imaging isocenter offset 50 cm)	[117]

**Table 2:** Summary of marker-based and marker-less FGPT systems discussed in the manuscript.

Author	Institution	Modality	Highlight	Ref
A. Commercial Marker-Based FGPT (Hitachi RGPT)				
Shimizu et al. 2014	Hokkaido University	Proton PBS	CTV coverage 48/48 vs. 9/48 free-breathing (HCC); ~50% liver dose reduction	[76]
Nishioka et al. 2024	Hokkaido University	Proton PBS	88.9% 5-yr bRFS for prostate cancer; ≥2 AE rate 8.9%	[104]
Chen et al. 2024	Johns Hopkins University	Proton PBS	Commissioning: dose delivery passed 3%/3 mm gamma; plan uncertainty within 2 mm	[105]
Tan et al. 2024	National Cancer Centre Singapore	Proton PBS	First RGPT-specific commissioning and QA report	[102]
Koh et al. 2025	National Cancer Centre Singapore	Proton PBS	Workflow FMEA	[106]
B. Clinical Marker-Less FGPT (NIRS Carbon-ion)				
Mori et al. 2016 and 2019; Hirai et al. 2016; Sakata et al. 2016 and 2020	NIRS (QST), Japan	Carbon-ion PBS	First marker-less gated PBS clinical trial for Carbon-ion therapy	[131,132,133,134,138]
C. NIRS Follow-Up AI Research for Marker-Less Tracking				
Hirai et al. 2019	NIRS (QST), Japan	Carbon-ion PBS	DNN tumor probability map	[139]
Hirai et al. 2020; Mori et al. 2020 and 2023; Takahashi et al. 2020	NIRS (QST), Japan	Carbon-ion PBS	Deep learning follow-up studies: regression models, synthetic fluoroscopy, patient-specific training	[140,141,142,143]

**Table 3:** Quantitative summary of AI-based registration and marker-less tracking methods, grouped by Section 4.2 paradigm taxonomy.

Ref	Author	Image	Speed on GPU	Highlight
A. Direct parameter/DVF regression				
[192]	de Vos 2017—DIRNet deformable	Cardiac cine MRI	<50 ms	Spatial transformer-based deformable registration.
[193]	de Vos 2019—DLIR (affine + deformable)	Cardiac MRI; Chest CT	<40 ms	Coarse-to-fine spatial transformer-based deformable image registration.
[194]	Li 2018	Brain MRI	~50 ms	Jointly optimize the spatial transformer and the fully convolutional network (FCN).
[195,196]	VoxelMorph (Balakrishnan 2018 and 2019)	Brain MRI	~24 s	UNet + STN.
[197]	CycleMorph (Kim 2021)	Brain MRI; Liver CECT	~1 s/pair	Topology regularization via cycle consistency.
[188]	DiffuseMorph (Kim 2022)	Brain MRI; Cardiac MRI	<1 s	Diffusion model for deformable registration. Iterative.
B. Segmentation-based				
[144]	Mylonas 2019	kV fluoroscopy, prostate	~9 ms	Prostate fiducial detection.
[198]	Roggen 2020	kV projection, spine SBRT	~0.5 s	ResNet, Mask R-CNN, Faster R-CNN. vertebra bone-based surrogate.
[199]	He 2022	kV projection (Varian), pancreas	~30 ms	Stent as a surrogate for pancreatic tumor motion. Perceptual Attention UNet.
[200]	Edmunds 2019	CBCT projections, lung	~0.5 s	Mask R-CNN; Diaphragm surrogate. Worse at lateral angles.
[139]	Hirai 2019	kV fluoroscopy, lung + liver Carbon-ion	<40 ms	4DCT-derived DRRs. Predict Target Probability Map (TPM).
[141]	Takahashi 2020	kV fluoroscopy, lung phantom	32.5 ms	Patient-specific FCN. Phantom proof of concept.
[201]	Terunuma 2018	kV fluoroscopy, lung	25 ms	“Importance recognition”: bone suppression.
[202]	Terunuma 2023	kV fluoroscopy, lung	8 ms	Attention heatmaps for explainability.
[203]	Huang 2024	Simulated kV, lung	170 ms/frame	Patient-specific Retina U-Net.
[204]	Mylonas 2025	kV projections, prostate	~10 ms	cGAN prostate segmentation. Trained on synthetic kV from planning data. Patient-specific model.
C. Image synthesis-based				
[205]	Lei 2020	kV proj → 3D CT, lung SBRT	<1 s/volume	TransNet GAN.
[206]	He 2021	kV projections, spine SBRT	~0.1 s	ResNetGAN spine-only decomposition to suppress soft tissue.
[207]	Fu 2023	kV projections, lung	~50 ms	Pix2Pix sDTI Target-only decomposed image suppresses anatomy.
[208]	Fu 2025	kV intra-fraction, lung	~50 ms	First clinical sDTI deployment.
[209]	Madden 2024	Simulated kV, pancreas SBRT	N/A	CBCT-DRR for better domain match for on-treatment tracking.
[210]	Ahmed 2025	kV intra-fraction, pancreas	~29 ms	cGAN CBCT-DRR fine-tuning.
[211,212]	Yan 2024/2025	Color fluoroscopy, lung	179.8 ms	DUCK-Net trained on DRRs.
D. Other methods				
[213]	Wang 2020	kV CBCT projections, lung	~20 ms	CRNN (CNN + RNN); RNN exploits the temporal continuity of projections.
[214]	Grama 2023	kV during VMAT, lung SBRT	~30 ms	Siamese network
[215]	Mok 2022	Brain MRI (atlas)	<0.1 s	ViT
[216]	Xu 2024	Stereoscopic kV (CyberKnife), lung	Real-time	Zero-shot Pre-trained DNN + template matching; uncertainty measure.

## Data Availability

No new data were created or analyzed in this study. Data sharing is not applicable to this article.

## Abbreviations

The following abbreviations are used in this manuscript:

AC	Abdominal Compression
ADC	Analog-to-Digital Converter
AI	Artificial Intelligence
a-Se	Amorphous Selenium
a-Si	Amorphous Silicon
a-Si:H	Hydrogenated Amorphous Silicon
BH	Breath Hold
CaW	Calcium Tungstate
CBCT	Cone Beam CT
CCD	Charge-Coupled Devices
CCTV	Closed-Circuit TV
CNN	Convolutional Neural Network
CsI	Cesium-Iodide
CsI:Na	Sodium activated cesium iodide
CsI:Tl	Thallium-Doped Cesium Iodide
CTV	Clinical Target Volume
DDCS	Dose Driven Continuous Scanning
DDPM	Denoising Diffusion Probabilistic Models
DE	Dual-Energy
DFPD	Dynamic Flat-Panel Detector
DM	Dose Monitor
DQE	Detective Quantum Efficiency
DRR	Digitally Reconstructed Radiograph
DVF	Deformation Vector Field
EBM	Energy Based Model
FCN	Fully Convolutional Network
FGPT	Fluoroscopy-Guided Particle Therapy
FMEA	Failure Modes and Effects Analysis
FOV	Field of View
FPD	Flat-Panel Detector
GAN	Generative Adversarial Networks
GNN	Graph Neural Network
HCL	Harvard Cyclotron Laboratory
HD95	95-Percentile Hausdorff Distance
HU	Hounsfield Unit
ICD	Interrupted Continuous Delivery
II	Image Intensifier
ITV	Internal Target Volume
JEPA	Joint Embedding Predictive Architecture
kV	Kilovoltage
Linac	Linear Accelerator
LLM	Large Language Model
MEE	Multiple Energy Extraction
MI	Mutual Information
MU	Monitor Units
MV	Megavoltage
NCC	Normalized Cross-Correlation
NeRF	Neural Radiance Fields
NIRS	National Institute of Radiological Sciences
PBS	Pencil Beam Scanning
PCR	Phase Controlled Rescanning
PS	Passive Scattering
PSI	Paul Scherrer Institute
PTV	Planning Target Volume
RGPT	Real-Time Gated Particle Therapy
RGSC	Respiratory Gating for Scanners
RNN	Recurrent Neural Network
RPM	Real-Time Position Management
RTRT	Real-Time Tumor-Tracking Radiotherapy
SBRT	Stereotactic Body Radiation Therapy
SEE	Single Energy Extraction
SEER	Surveillance, Epidemiology, and End Results
SGRT	Surface Guided Radiation Therapy
SOBP	Spread-Out Bragg Peak
SSD	Sum of Squared Differences
STN	Spatial Transformer Network
TCP	Tumor Control Probability
TFT	Thin-Film Transistor
TPM	Tumor Probability Map
TRE	Tracking Registration Error
ViT	Vision Transformer
WEPL	Water Equivalent Path Length
ZnCdS	Zinc-Cadmium-Sulfide
ZnCdS:Ag	Silver-Activated Zinc-Cadmium-Sulfide
